# Supplementary material for: Smoking prevalence and purchasing of menthol cigarettes since the menthol flavour ban in Great Britain: a population-based survey between 2020 and 2023
Source: Tob Control. 2024 Mar 12;34(4):e058390. doi: 10.1136/tc-2023-058390 (PMC12322387; doi:10.1136/tc-2023-058390)
Supplement: online supplemental file 1 [file tc-34-4-s001.pdf]

SUPPLEMENT

Supplementary to article: “Smoking prevalence and purchasing of menthol cigarettes since the menthol flavour ban in Great Britain: a population-based survey between 2020 and 2023“

1. Missing values

Table S1: Proportion of data missing for each variable (N = 67,746).

Variable	*Missing values, n (%)*
Age	0 (0)
Gender	110 (0.2)
Nation	0 (0)
Social grade	0 (0)
Cigarette smoking	563 (0.8)
Flavoured cigarettes (cigarette smoking n = 9,195)	208 (2.3)
Source of purchase (cigarette smoking n = 6,757) *	191 (2.8)

* Question about source of purchase not asked in England in May, June, August, September, November, and December 2022, and in February and March 2023. Therefore, these months were excluded for this question.

2. Sensitivity analysis: unweighted

Table S2: Characteristics of survey respondents between October 2020 and March 2023, unweighted vs. weighted (N = 66,868).

Characteristic	*Unweighted*	*Weighted*
Age, median (IQR)	52 (35-66)	49 (33-63)
Gender, n (%)		
Female	34108 (51.0)	33918 (50.8)
Male	32360 (48.4)	32427 (48.6)
Non-binary	400 (0.6)	400 (0.6)
Social grade, n (%)		
ABC1	43808 (65.5)	37341 (55.9)
C2DE	23060 (34.5)	29404 (44.1)
Nation, n (%)		
England	47641 (71.2)	57628 (86.3)
Scotland	12442 (18.6)	5842 (8.8)
Wales	6785 (10.1)	3275 (4.9)
Smoking cigarettes, n (%)	8965 (13.4)	9773 (14.6)

Table S3: Prevalence of menthol cigarette smoking in different population groups who smoke cigarettes (unmodelled, weighted).

Quarter (Q)	*Great Britain, all adults, % (95% CI)*	*Great Britain, 18-to-24-year-olds, % (95% CI)*	*England, all adults, % (95% CI)*	*Scotland, all adults, % (95% CI)*	*Wales, all adults, % (95% CI)*
Q4 2020	17.5 (14.8-20.3)	24.5 (16.7-32.3)	17.7 (14.6-20.8)	11.0 (5.0-17.0)	23.5 (13.9-33.2)
Q1 2021	16.7 (14.0-19.4)	30.4 (21.4-39.3)	17.0 (14.0-20.0)	11.8 (7.1-16.5)	19.6 (10.8-28.4)
Q2 2021	12.7 (10.2-15.2)	15.9 (9.6-22.3)	12.0 (9.3-14.8)	15.3 (9.5-21.0)	22.0 (11.0-32.9)
Q3 2021	14.9 (12.2-17.6)	14.4 (8.1-20.7)	15.7 (12.6-18.8)	9.4 (4.9-13.9)	10.8 (3.0-18.7)
Q4 2021	16.2 (13.5-19.0)	18.9 (11.0-26.9)	16.6 (13.4-19.7)	9.8 (5.4-14.2)	24.4 (13.5-35.3)
Q1 2022	18.7 (15.7-21.7)	26.7 (17.9-35.5)	20.0 (16.6-23.4)	10.2 (5.2-15.1)	11.6 (5.1-18.0)
Q2 2022	13.9 (11.2-16.6)	15.8 (9.4-22.2)	14.4 (11.3-17.4)	10.9 (5.2-16.7)	10.9 (4.1-17.7)
Q3 2022	14.0 (11.5-16.6)	15.3 (8.8-21.8)	13.8 (11.0-16.6)	15.9 (9.7-22.0)	15.5 (7.9-23.1)
Q4 2022	15.0 (12.2-17.7)	15.9 (9.2-22.7)	15.6 (12.5-18.7)	11.6 (6.4-16.9)	7.5 (2.2-12.8)
Q1 2023	13.0 (10.4-15.6)	22.4 (13.6-31.1)	13.6 (10.7-16.6)	8.8 (3.9-13.8)	7.7 (1.1-14.4)

Table S4: Prevalence of menthol cigarette smoking among people who smoke cigarettes (unmodelled, unweighted).

Quarter (Q)	***Great Britain, all adults, % (95% CI)***	***Great Britain, 18-to-24-year-olds, % (95% CI)***	***England, all adults, % (95% CI)***	***Scotland, all adults, % (95% CI)***	***Wales, all adults, % (95% CI)***
Q4 2020	17.6 (15.1-20.1)	25.4 (18.1-32.6)	18.3 (15.4-21.3)	10.3 (5.1-15.5)	23.3 (14.4-32.2)
Q1 2021	16.7 (14.3-19.0)	29.0 (21.1-36.9)	17.5 (14.6-20.4)	12.6 (7.9-17.4)	18.9 (10.9-27)
Q2 2021	13.0 (10.8-15.2)	20.0 (13.3-26.7)	11.6 (9.2-14.1)	16.0 (10.3-21.8)	18.4 (9.5-27.3)
Q3 2021	13.7 (11.4-15.9)	16.9 (10.4-23.5)	15.1 (12.4-17.9)	9.9 (5.4-14.4)	10.0 (3.3-16.7)
Q4 2021	15.6 (13.2-18.0)	18.9 (11.5-26.3)	15.8 (13.0-18.6)	11.4 (6.6-16.3)	22.2 (13.0-31.5)
Q1 2022	16.6 (14.2-19.0)	24.6 (17.1-32.1)	19.2 (16.1-22.2)	9.9 (5.5-14.3)	12.5 (6.0-19.0)
Q2 2022	12.9 (10.7-15.2)	17.8 (11.2-24.3)	13.7 (11.0-16.4)	10.8 (5.6-16)	11.0 (4.4-17.5)
Q3 2022	14.9 (12.6-17.2)	16.1 (10.0-22.2)	14.5 (11.8-17.2)	15 (9.7-20.4)	17.4 (9.5-25.3)
Q4 2022	14.6 (12.2-16.9)	18.1 (11.3-24.9)	15.9 (13.0-18.8)	12.4 (7.3-17.6)	9.2 (3.0-15.4)
Q1 2023	12.3 (10.1-14.6)	20.5 (12.9-28.1)	13.6 (10.9-16.3)	9.6 (4.6-14.6)	7.2 (1.5-12.9)

Table S5: Sources of cigarette purchases in the last six months (not mutually exclusive) among those who smoke cigarettes between October 2020 and March 2023 (unweighted).

Source of purchase	***Among people smoking menthol cigarettes, n (%)***	***Among people smoking non-flavoured cigarettes, n (%)***	***P-value[1]***
Newsagent\Off licence\Corner shop	716 (71.3)	3755 (68.6)	0.096
Petrol garage shop	417 (41.5)	2140 (39.1)	0.157
Supermarket	725 (72.2)	4007 (73.2)	0.535
Cash and Carry	57 (5.7)	256 (4.7)	0.201
Internet	25 (2.5)	145 (2.6)	0.855
Bar in pub	19 (1.9)	69 (1.3)	0.150
Other sources	6 (0.6)	57 (1.0)	0.325
Illicit			
Newsagent\Off license\Corner shop – “under the counter”	75 (7.5)	294 (5.4)	0.01
Friends	54 (5.4)	272 (5.0)	0.641
Trusted local	34 (3.4)	176 (3.2)	0.854
Person in pub	24 (2.4)	86 (1.6)	0.087
Person on the street	21 (2.1)	113 (2.1)	1.000
Vending machine pub	9 (0.9)	41 (0.7)	0.769
Cross-border			
Buy them from abroad and bring them back	108 (10.8)	539 (9.8)	0.409
Friends/family bring from abroad	2 (0.2)	7 (0.1)	0.923

[1]All values for Cramer’s V were ≤0.10.

Table S6: Sources of cigarette purchases in the last six months (not mutually exclusive) by nation between October 2020 and March 2023 (unweighted).

Source of purchase	***Among people smoking menthol cigarettes in England, n (%)***	***Among people smoking menthol cigarettes in Scotland, n (%)***	***Among people smoking menthol cigarettes in Wales, n (%)***	***P-value[1]***
Newsagent/Off licence/Corner shop	546 (71.9)	98 (69.0)	72 (69.9)	0.737
Petrol garage shop	315 (41.5)	56 (39.4)	46 (44.7)	0.715
Supermarket	545 (71.8)	102 (71.8)	78 (75.7)	0.702
Cash and Carry	44 (5.8)	9 (6.3)	4 (3.9)	0.686
Internet	17 (2.2)	6 (4.2)	2 (1.9)	0.353
Bar in pub	16 (2.1)	2 (1.4)	1 (1.0)	0.657
Other sources	5 (0.7)	1 (0.7)	0 (0.0)	0.722
Illicit				
Newsagent/Off license/ Corner shop – “under the counter”	60 (7.9)	5 (3.5)	10 (9.7)	0.125
Friends	44 (5.8)	3 (2.1)	7 (6.8)	0.162
Trusted local	29 (3.8)	0 (0.0)	5 (4.9)	0.048
Person in pub	20 (2.6)	3 (2.1)	1 (1.0)	0.568
Person on the street	16 (2.1)	3 (2.1)	2 (1.9)	0.994
Vending machine pub	8 (1.1)	1 (0.7)	0 (0.0)	0.548
Cross-border				
Buy them from abroad and bring them back	83 (10.9)	16 (11.3)	9 (8.7)	0.778
Friends/family bring from abroad	1 (0.1)	0 (0.0)	1 (1.0)	0.170

[1]All values for Cramer’s V were ≤0.10.

3. Sensitivity analysis: using all flavoured cigarettes instead of only menthol cigarettes

Table S7: Prevalence of flavoured cigarette smoking in different population groups who smoke cigarettes (unmodelled, weighted).

Quarter (Q)	*Great Britain, all adults, % (95% CI)*	*Great Britain, 18-to-24-year-olds, % (95% CI)*	*England, all adults, % (95% CI)*	*Scotland, all adults, % (95% CI)*	*Wales, all adults, % (95% CI)*
Q4 2020	20.1 (17.1-23.1)	25.7 (17.8-33.6)	20.5 (17.2-23.8)	11.6 (5.5-17.7)	25.0 (15.3-34.8)
Q1 2021	18.8 (16.0-21.6)	34.8 (25.6-43.9)	18.9 (15.7-22.0)	16.7 (11.0-22.4)	21.9 (12.9-30.9)
Q2 2021	15.7 (13.0-18.4)	19.7 (12.6-26.8)	15.1 (12.1-18.1)	18.2 (11.5-24.9)	24.4 (13.2-35.5)
Q3 2021	17.0 (14.2-19.8)	17.1 (10.3-23.9)	17.8 (14.6-21.0)	11.2 (6.3-16.2)	14.0 (5.1-22.8)
Q4 2021	18.2 (15.3-21.1)	24.1 (15.2-33.1)	18.5 (15.2-21.8)	11.6 (6.7-16.5)	25.5 (14.5-36.5)
Q1 2022	21.4 (18.2-24.6)	30.8 (21.6-40.0)	23.0 (19.3-26.6)	12.0 (6.8-17.2)	12.1 (5.6-18.6)
Q2 2022	15.6 (12.8-18.4)	17.1 (10.5-23.7)	15.9 (12.7-19.0)	13.5 (7.3-19.8)	14.1 (6.6-21.7)
Q3 2022	16.9 (14.2-19.7)	18.8 (11.6-26.0)	16.8 (13.7-19.9)	17.6 (11.3-23.8)	18.2 (10.1-26.3)
Q4 2022	17.6 (14.7-20.6)	16.8 (10.0-23.7)	18.2 (14.9-21.5)	13.5 (7.7-19.3)	12.8 (5.7-20.0)
Q1 2023	14.3 (11.6-17.0)	26.4 (17.1-35.7)	15.0 (12.0-18.1)	8.8 (3.9-13.8)	10.1 (2.3-17.9)

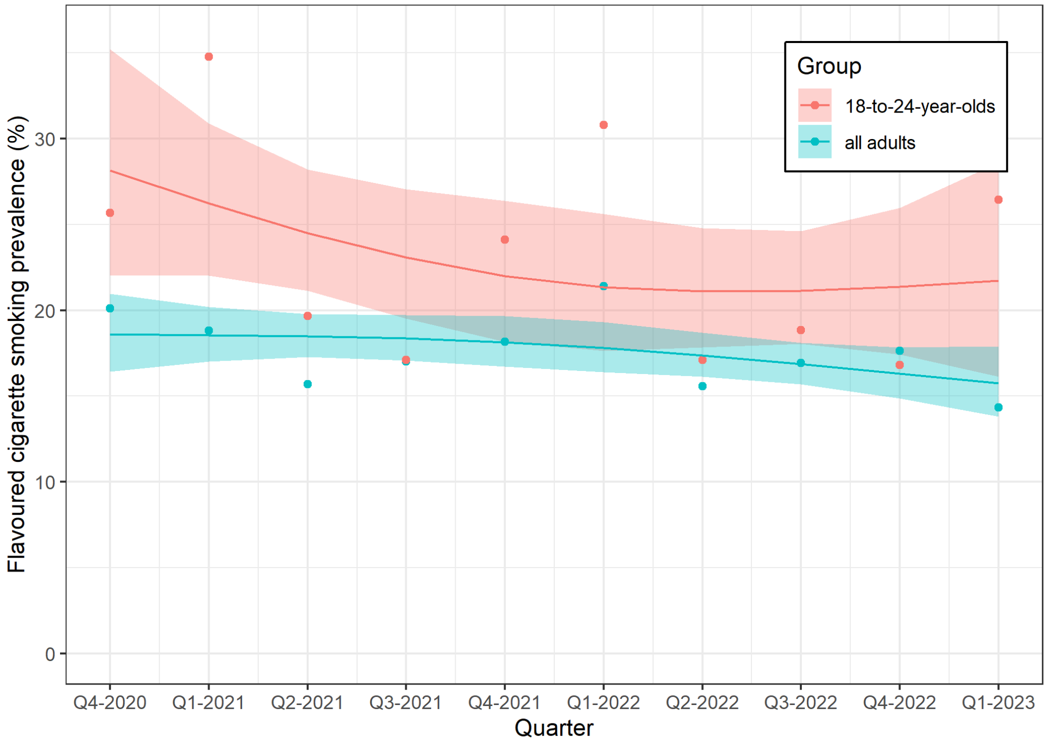

Figure S1: Weighted prevalence of smoking flavoured cigarettes among all adults who smoke cigarettes and those aged 18-24 years in Great Britain over time. Lines and shaded bands represent point estimates and 95% compatibility intervals, respectively, from logistic regression with time modelled with restricted cubic splines (3 knots). The points represent unmodelled data.

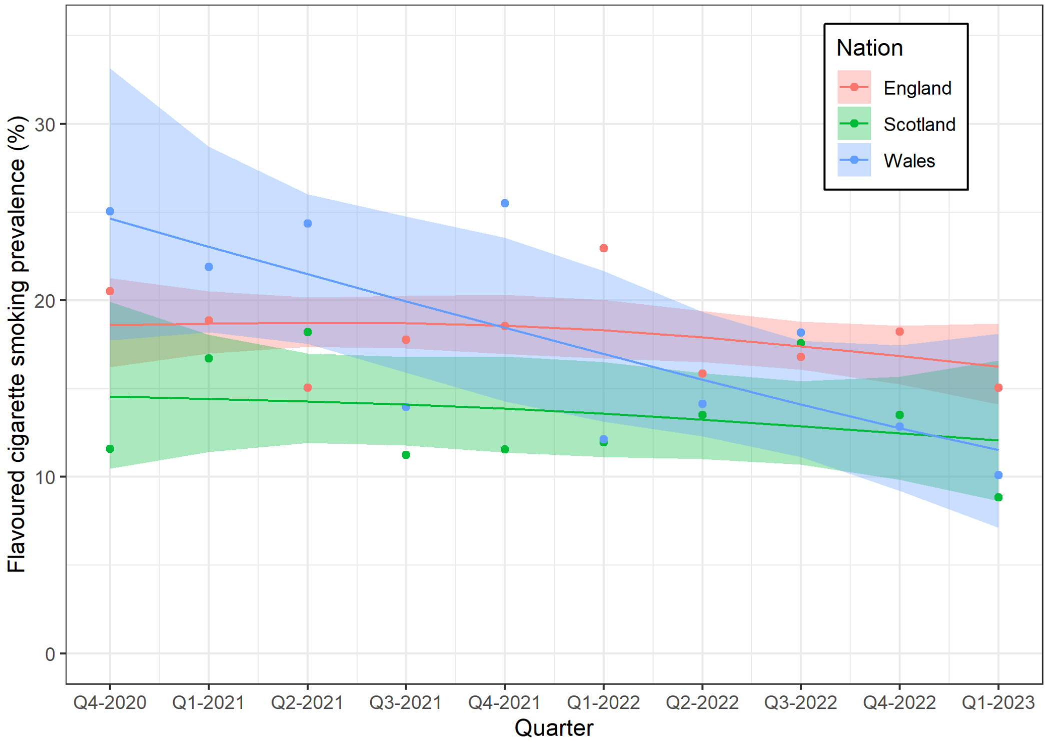

Figure S2: Weighted prevalence of smoking flavoured cigarettes among adults smoking cigarettes in England, Scotland, and Wales over time. Lines and shaded bands represent point estimates and 95% compatibility intervals, respectively, from logistic regression with time modelled with restricted cubic splines (3 knots). The points represent unmodelled data.

Table S8: Modelled prevalence and prevalence ratios comparing first (Q4 2020) to last quarter (Q1 2023) (weighted).

Sample	***Menthol flavour***			***Any flavour***		
	Q4 2020, % (95% CI)	Q1 2023, % (95% CI)	PR (95% CI)	Q4 2020, % (95% CI)	Q1 2023, % (95% CI)	PR (95% CI)
All adults	16.2 (14.1-18.4)	13.7 (11.9-15.8)	0.85 (0.71-1.01)	18.6 (16.4-21.0)	15.7 (13.8-17.9)	0.85 (0.72-0.99)
18-to-24-year-olds	25.7 (19.7-32.8)	19.4 (11.9-26.0)	0.75 (0.63-0.89)	28.1 (22.0-35.2)	21.7 (13.8-28.6)	0.77 (0.61-0.98)
Adults in England	16.2 (13.9-18.7)	14.2 (12.2-16.5)	0.88 (0.72-1.06)	18.6 (16.2-21.3)	16.3 (14.1-18.7)	0.87 (0.73-1.05)
Adults in Scotland	12.0 (8.3-17.0)	11.3 (8.0-15.8)	0.94 (0.59-1.53)	14.6 (10.5-19.9)	12.1 (8.6-16.6)	0.83 (0.53-1.31)
Adults in Wales	22.5 (15.8-31.0)	8.1 (4.7-13.7)	0.36 (0.19-0.62)	24.6 (17.8-33.1)	11.5 (7.1-18.1)	0.47 (0.27-0.79)
Adults in Scotland vs. England	NA	NA	1.07 (0.64-1.80)	NA	NA	0.95 (0.58-1.55)
Adults in Wales vs. England	NA	NA	0.41 (0.21-0.75)	NA	NA	0.54 (0.30-0.93)

Table S9: Purchase sources in the last six months (not mutually exclusive) among those smoking flavoured vs non-flavoured cigarettes between October 2020 and March 2023 (weighted).

Source of purchase	***Among people smoking flavoured cigarettes, % (95% CI)***	***Among people smoking non-flavoured cigarettes, % (95% CI)***	***P-value[1]***
Newsagent\Off licence\Corner shop	72.4 (69.4-75.3)	69.8 (68.4-71.1)	0.078
Petrol garage shop	42.7 (39.4-46)	40.9 (39.3-42.4)	0.251
Supermarket	72.3 (69.3-75.2)	72.2 (70.8-73.5)	0.935
Cash and Carry	6.5 (4.7-8.2)	5.3 (4.5-6.0)	0.104
Internet	2.6 (1.5-3.7)	2.7 (2.2-3.2)	0.857
Bar in pub	1.8 (0.9-2.8)	1.3 (0.9-1.6)	0.157
Other sources	0 (0.0-0.0)	0.9 (0.7-1.2)	0.127
Illicit			
Newsagent\Off license\Corner shop – “under the counter”	8.1 (6.2-10.0)	6.0 (5.3-6.8)	0.009
Friends	5.8 (4.2-7.3)	5.5 (4.8-6.3)	0.764
Trusted local	3.6 (2.3-4.9)	3.5 (2.9-4.1)	0.882
Person in pub	2.8 (1.6-3.9)	1.8 (1.4-2.2)	0.027
Person on the street	2.4 (1.4-3.4)	2.3 (1.8-2.8)	0.791
Vending machine pub	1.1 (0.3-1.9)	0.9 (0.6-1.2)	0.452
Cross-border			
Buy them from abroad and bring them back	11.3 (9.1-13.5)	9.8 (8.9-10.7)	0.114
Friends/family bring from abroad	0.2 (0.0-0.6)	0.1 (0.0-0.2)	0.327

[1]All values for Cramer’s V were ≤0.10.

Table S10: Type of purchase in the last six months (not mutually exclusive) among those smoking flavoured cigarettes by nation between October 2020 and March 2023 (weighted).

Type of purchase	***Among people smoking flavoured cigarettes, % (95% CI)***	***Among people smoking non-flavoured cigarettes, % (95% CI)***
Licit	93.6 (92.0-95.2)	93.5 (92.7-94.2)
Illicit	14.7 (12.3-17.1)	12.5 (11.4-13.5)
Cross-border	11.5 (9.4-13.7)	9.9 (9.0-10.8)

Table S11: Purchase sources in the last six months (not mutually exclusive) among those smoking flavoured cigarettes by nation between October 2020 and March 2023 (weighted).

Source of purchase	*Among people smoking flavoured cigarettes in England, % (95% CI)*	*Among people smoking menthol cigarettes in Scotland, % (95% CI)*	*Among people smoking menthol cigarettes in Wales, % (95% CI)*	*P-value[1]*
Newsagent/Off licence/Corner shop	72.9 (69.7-76.0)	67.3 (59.4-75.3)	70.4 (61.3-79.5)	0.561
Petrol garage shop	42.2 (38.6-45.8)	42.4 (34.1-50.7)	47.4 (37.4-57.4)	0.733
Supermarket	72.0 (68.7-75.2)	72.1 (64.6-79.7)	76.6 (68.2-84.9)	0.741
Cash and Carry	6.6 (4.8-8.5)	7.6 (2.9-12.2)	4.2 (0.1-8.2)	0.709
Internet	2.7 (1.6-3.9)	3.3 (0.8-5.7)	3.1 (0.0-6.8)	0.956
Bar in pub	2.1 (1.1-3.2)	1.3 (0.0-3.4)	0.6 (0.0-1.8)	0.638
Other sources	0.5 (0.0-1.0)	0.4 (0.0-1.1)	0.0 (0.0-0.0)	0.844
Illicit				
Newsagent/Off license/Corner shop – “under the counter”	8.5 (6.4-10.6)	5.3 (1.5-9.2)	9.5 (3.4-15.6)	0.607
Friends	6.2 (4.4-7.9)	6.1 (1.3-10.9)	4.6 (1.1-8.2)	0.888
Trusted local	3.9 (2.5-5.3)	2.8 (0.0-6.3)	5.1 (0.7-9.5)	0.801
Person in pub	2.9 (1.7-4.2)	4.9 (0.5-9.4)	1.7 (0.0-4.9)	0.524
Person on the street	2.4 (1.4-3.5)	4.9 (0.6-9.1)	3.1 (0.0-6.9)	0.444
Vending machine pub	1.1 (0.3-1.9)	1.1 (0.0-3.2)	1.7 (0.0-4.9)	0.925
Cross-border				
Buy them from abroad and bring them back	11.5 (9.2-13.9)	14.1 (8.0-20.2)	8.9 (3.4-14.4)	0.646
Friends/family bring from abroad	0.2 (0.0-0.6)	0.0 (0.0-0.0)	1.1 (0.0-3.3)	0.323

[1]All values for Cramer’s V were ≤0.10.

4. Sensitivity analysis: prevalence of smoking menthol cigarettes as a proportion of all adults (or all 18-to-24-year-olds)

Table S12: Prevalence of cigarette smoking among all participants in different population groups (unmodelled, weighted).

Quarter (Q)	*Great Britain, all adults, % (95% CI)*	*Great Britain, 18-24-year-olds, % (95% CI)*	*England, all adults, % (95% CI)*	*Scotland, all adults, % (95% CI)*	*Wales, all adults, % (95% CI)*
Q4 2020	2.6 (2.1-3.0)	5.5 (3.5-7.4)	2.6 (2.1-3.1)	1.3 (0.6-2.1)	3.7 (2.0-5.4)
Q1 2021	2.5 (2.1-2.9)	6.0 (3.9-8.1)	2.6 (2.1-3.1)	1.8 (1.0-2.5)	2.8 (1.4-4.1)
Q2 2021	1.9 (1.5-2.3)	4.1 (2.4-5.8)	1.9 (1.4-2.3)	2.2 (1.3-3.1)	2.7 (1.2-4.2)
Q3 2021	2.2 (1.7-2.6)	2.8 (1.5-4.1)	2.3 (1.8-2.7)	1.4 (0.7-2.1)	1.6 (0.4-2.8)
Q4 2021	2.4 (1.9-2.8)	3.5 (1.9-5.1)	2.4 (1.9-2.8)	1.7 (0.9-2.5)	3.5 (1.7-5.3)
Q1 2022	2.7 (2.3-3.2)	5.1 (3.2-7.1)	2.9 (2.4-3.5)	1.5 (0.7-2.2)	2.0 (0.8-3.1)
Q2 2022	2.0 (1.6-2.4)	2.9 (1.7-4.2)	2.1 (1.6-2.5)	1.5 (0.6-2.3)	1.7 (0.6-2.9)
Q3 2022	2.1 (1.7-2.5)	3.2 (1.7-4.6)	2.1 (1.7-2.6)	2.1 (1.2-3.0)	2.2 (1.1-3.4)
Q4 2022	2.1 (1.7-2.6)	2.8 (1.6-4.1)	2.3 (1.8-2.8)	1.5 (0.8-2.2)	1.0 (0.3-1.6)
Q1 2023	1.8 (1.4-2.2)	4.2 (2.4-6.0)	1.9 (1.5-2.4)	1.1 (0.5-1.7)	1.2 (0.1-2.2)

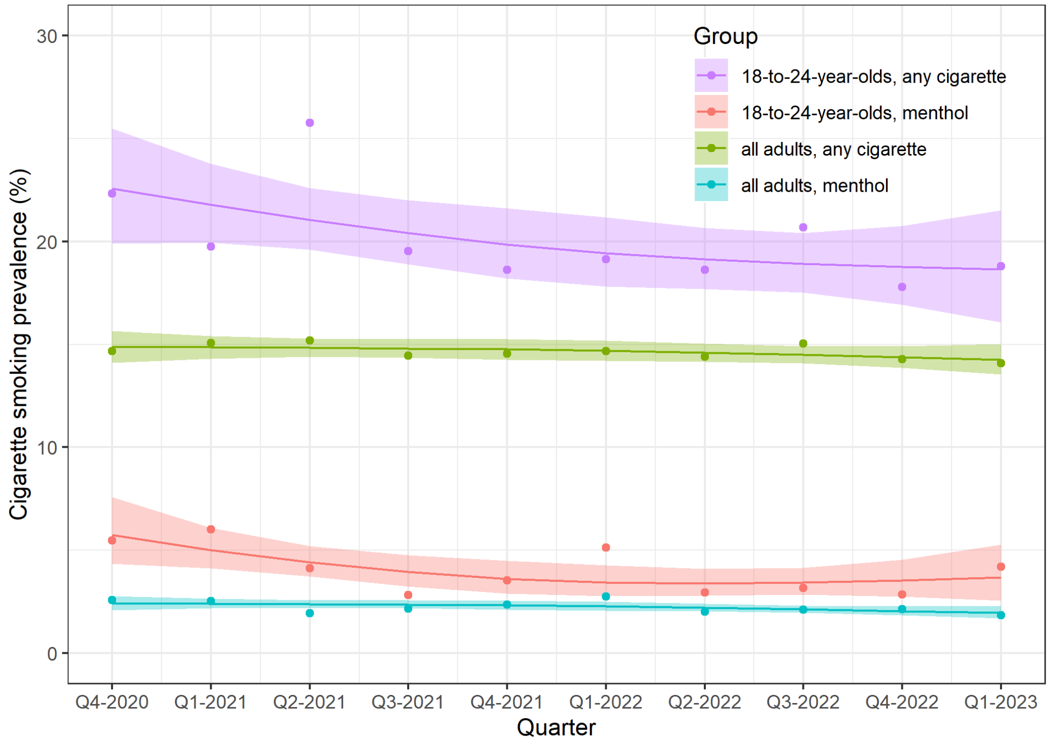

Figure S3: Weighted prevalence of smoking any type of cigarette and smoking menthol cigarettes among all adults and those aged 18-24 years in Great Britain over time. Lines and shaded bands represent point estimates and 95% compatibility intervals, respectively, from logistic regression with time modelled with restricted cubic splines (3 knots). The points represent unmodelled data.

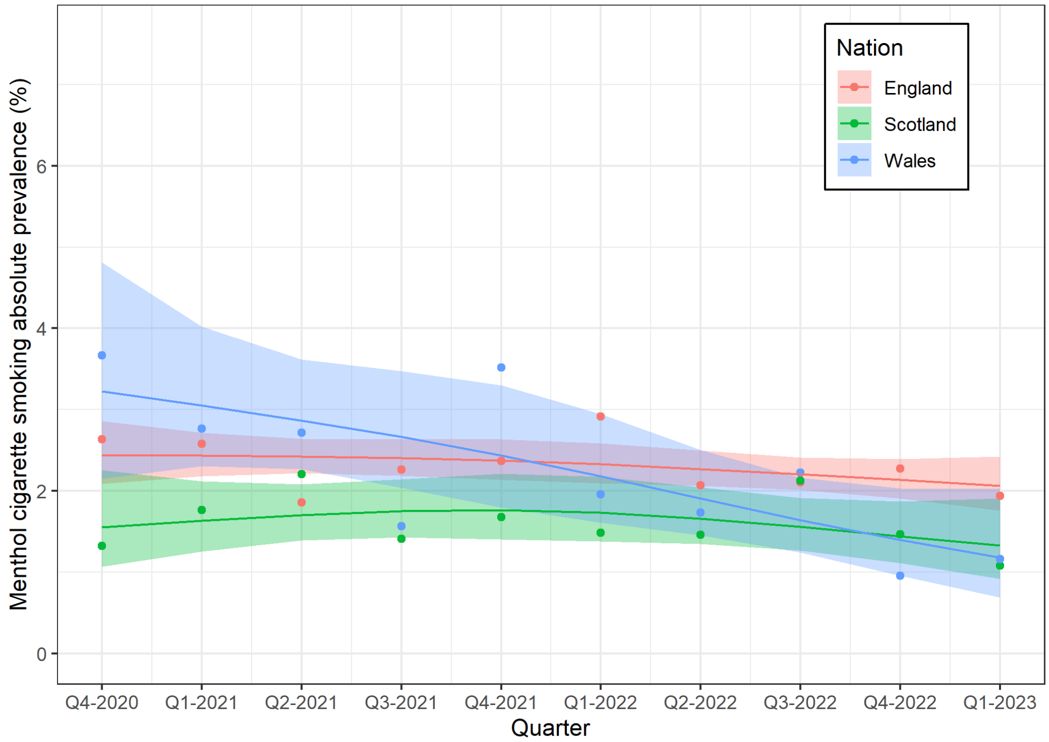

Figure S4: Weighted prevalence of smoking flavoured cigarettes among adults in England, Scotland, and Wales over time. Lines and shaded bands represent point estimates and 95% compatibility intervals, respectively, from logistic regression with time modelled with restricted cubic splines (3 knots). The points represent unmodelled data.

Table S13: Modelled prevalence and prevalence ratios comparing first (Q4 2020) to last quarter (Q1 2023) (weighted), comparing prevalence (i.e., among all who smoke) to prevalence among all participants.

Sample	***Prevalence among those who smoke***			***Prevalence among all participants***		
	Q4 2020, % (95% CI)	Q1 2023, % (95% CI)	PR (95% CI)	Q4 2020, % (95% CI)	Q1 2023, % (95% CI)	PR (95% CI)
All adults	16.2 (14.1-18.4)	13.7 (11.9-15.7)	0.85 (0.71-1.01)	2.4 (2.1-2.8)	2.0 (1.7-2.3)	0.81 (0.68-0.98)
18-to-24-year-olds	25.7 (19.7-32.8)	19.4 (14.1-26.0)	0.75 (0.63-0.90)	5.7 (4.3-7.6)	3.7 (1.7-5.3)	0.64 (0.53-0.77)
Adults in England	6.2 (13.9-18.7)	14.2 (12.2-16.5)	0.88 (0.73-1.06)	2.4 (2.1-2.9)	2.1 (1.8-2.4)	0.84 (0.69-1.04)
Adults in Scotland	12.0 (8.3-17.0)	11.3 (7.9-15.8)	0.94 (0.58-1.50)	1.6 (1.1-2.3)	1.3 (0.9-1.9)	0.85 (0.50-1.38)
Adults in Wales	22.5 (15.8-31.0)	8.1 (4.6-13.7)	0.36 (0.19-0.62)	3.2 (2.2-4.8)	1.2 (0.7-2.0)	0.37 (0.19-0.68)
Adults in Scotland vs. England	NA	NA	1.07 (0.63-1.79)	NA	NA	1.00 (0.57-1.66)
Adults in Wales vs. England	NA	NA	0.41 (0.20-0.71)	NA	NA	0.43 (0.22-0.82)
